# Mushroom Poisoning-Related Cardiac Toxicity: A Case Report and Systematic Review

**DOI:** 10.3390/toxins16060265

**Published:** 2024-06-10

**Authors:** Giuseppe Balice, Maxime Boksebeld, Quentin Barrier, Sara Boccalini, Behrouz Kassai-Koupai, Nathalie Paret, Guillaume Grenet

**Affiliations:** 1 Service Hospitalo-Universitaire de Pharmacotoxicologie, Hospices Civils de Lyon, 69003 Lyon, France; maxime.boksebeld@chu-lyon.fr (M.B.); behrouz.kassai-koupai@chu-lyon.fr (B.K.-K.); nathalie.paret@chu-lyon.fr (N.P.); guillaume.grenet@chu-lyon.fr (G.G.); 2 Fédération de Cardiologie Médicale, Hôpital Louis Pradel, Hospices Civils de Lyon, 69500 Bron, France; 3 Service de Radiologie Cardiovasculaire et Thoracique, Hôpital Louis Pradel, Hospices Civils de Lyon, 69500 Bron, France; 4 Centre d’Investigation Clinique de Lyon (CIC1407 INSERM), Hospices Civils de Lyon, 69500 Bron, France; 5 Laboratoire de Biométrie et Biologie Evolutive UMR 5558, Université Lyon 1, CNRS, 69100 Villeurbanne, France

**Keywords:** mushroom, poisoning, troponin, ECG, myocarditis, cardiac toxicity, orellanian syndrome, cortinarius

## Abstract

We encountered a case of mushroom intoxication complicated by “toxic-like” myocarditis. Because of the lack of systematized knowledge on this subject, we performed a systematic review of the literature on cardiac toxicity in mushroom poisoning (MP). The aim of this study was to identify and describe the severity, the causal relationship, and the mushroom species involved in other reported cardiac events associated with MP. We included 39 studies in our review. We found 106 cases of cardiac events associated with MP, including 18 deaths. A wide variety of cardiac manifestations were reported, ranging from the simple elevation of cardiac enzymes (n = 61) to ventricular tachycardia (n = 14), acute heart failure (n = 18), and myocarditis (n = 7). Causal relationship between cardiac manifestations and mushroom poisoning was assessed for 42 patients, applying the algorithm validated by the French Toxicovigilance Coordination Committee. Twenty-three cases (54.8%) had a “possible” causal relationship, eight cases (19%) a “probable” relationship, and ten cases (23.8%) a “very probable” relationship. Several fungal genera were involved in reported cases, including *Amanita* but also rarer ones like *Russula* and *Tricholoma*. In conclusion, we showed that cases of cardiac toxicity following MP have been documented in the existing literature, and for some of them, we assessed a strong causal relationship.

## 1. Introduction

Despite being an ancient and appreciated cooking ingredient in many countries of the world, only a few mushroom species are edible, and sometimes, they may be barely distinguishable from toxic ones by the inexpert gatherer. The rate of reported fatal mushroom poisoning (MP) has been increasing worldwide in recent decades [1], with regional differences. At the beginning of 2000s, between 50 and 100 fatal cases/year were reported in Western Europe [2]. Conversely, in a recent retrospective study on the US National Poison Data System, a mean of 7428 MP cases/year have been reported in the 1999–2016 period, with a low lethality rate (less than 3 lethal cases/year) [3]. However, MP incidence data are surely affected by an underreporting bias. For instance, the American Association of Poison Control Centers (PCCs) estimates that only 5% of annual fatal poisonings are reported [4].

In this context of underreporting, only the most frequent toxidromes have been described and classified. The classification of MP syndromes proposed by Rumack and Spoerke in 1994 [5] was then developed by including newly discovered toxidromes [6]. Described MP syndromes involve the gastro-intestinal tract, the liver, the kidney, the nervous system, or the muscles. Nonetheless, cardiac toxicity is not mentioned. A new classification has been recently proposed by White et al. [7]. They reported several cardiac symptoms across some of the mushroom poisoning groups. In particular, they reported the risk of cardiac arrhythmias and myocardial ischemia in the “Group 2A—hallucinogenic mushrooms”, and the risk of acute myocarditis and cardiac failure in the “Group 3B—delayed onset myotoxicity”. However, the diagnostic algorithm which they proposed did not include any assessment of the possible cardiac toxicity.

In this context of lacking evidence, we encountered the case of a woman presenting with orellanian syndrome followed by myocarditis in our clinical practice at Lyon PCC. The cardiac MRI had a “toxic-like” pattern, and all common myocarditis causes were ruled out at differential diagnosis. We therefore hypothesized that myocarditis may be a direct consequence of MP (see below for the detailed case report). Looking at the literature for a confirmation of such a hypothesis, we found only sparse and unsystematized knowledge on this subject: both Ponsiglione et al. and Vallianou et al. presented a case report of myocarditis following MP, but they pointed out the scarcity of pre-existent literature [8,9].

Thus, we decided to perform a systematic literature review. Our main aim was to describe clinical manifestations of all reported MP cases related to cardiac toxicity. Secondarily, we aimed to describe their severity, the strength of their causal relationship, and the mushroom species involved in the poisoning.

### Case Report

A 44-year-old woman, with no reported medical history, no long-course medications, and, notably, without any previous cardiac complaint, presented vomiting and diarrhea few hours after mushroom consumption. Four days later, she presented abdominal pain and anuria, and thus, she was referred to our accident and emergency department. Plasma creatinine and urea were 1000 µmol/L and 25 mmol/L, respectively. Hepatic enzymes were normal. Due to renal failure and compensated metabolic acidosis, she was transferred to the intensive care unit, and dialysis was started for her three times a week. Urinary and plasmatic orellanine analyses were performed 5 days after mushroom consumption. Orellanine was detected in plasma and urine (the limit of detection for such biomatrices is 0.1 ng/mL), but its concentration was below the limit of quantitation (0.5 ng/mL). Fifteen days after the exposure, our patient was still anuric, plasma creatinine being 670 µmol/L. Twenty-three days after the exposure, diuresis restarted at a 150–200 mL/24 h rate. Twenty-seven days after exposure, our patient was discharged, but the dialysis schedule was kept unchanged. Thirty-four days after exposure, she presented anemia (blood hemoglobin dosage: 60 g/L), thus requiring iron supplementation, erythropoietin injections, and then blood transfusion. Forty days after exposure, the patient presented respiratory distress and was hospitalized in the intensive care unit due to bilateral pleural effusion. Left-ventricle ejection fraction (LVEF) was 20% with diffuse cardiac hypokinesia. Troponin-I concentration was 327 ng/L, and brain natriuretic peptide (BNP) was 3612 ng/L. Electrocardiogram was normal. Coronarography was not performed, and coronary computed tomography (CT) scan was normal. Forty-eight days after exposure, cardiac enzymes reverted to normal values, but the echocardiogram still detected global hypokinesia of the left ventricle, with an unsatisfactory LVEF (25%).

At this point, a cardiac magnetic resonance imaging (MRI) was performed, showing diffuse oedema of the left ventricle and pericardial enhancement. Such findings were not consistent with silent ischemia caused by repeated hemodialysis (“myocardial hibernation”). The cardio-radiologist rather retained the diagnosis of myopericarditis and judged the imaging pattern compatible with a toxic injury. Rheumatoid factor, anti-nucleus antibodies, and antineutrophil cytoplasmic antibodies tested negative, and there was no abnormal consumption of complement, thus excluding autoimmune etiology for the myopericarditis. All serological tests for B19 parvovirus, HHV6, HIV, HBV, and HCV were negative, and serologies for CMV and EBV were positive for IgG only, thus excluding a viral etiology for the myopericarditis. Bisoprolol, valsartan/sacubitril, and spironolactone were initiated. Fifty-three days after exposure, the patient was discharged with 40% LVEF. Eighty-nine days after exposure, LVEF was 50%. Eight months after exposure, our patient was still on dialysis and her plasma creatinine was 500 µmol/L. She was enlisted for kidney transplantation.

## 2. Results

### 2.1. Literature Search

**Figure A1:**
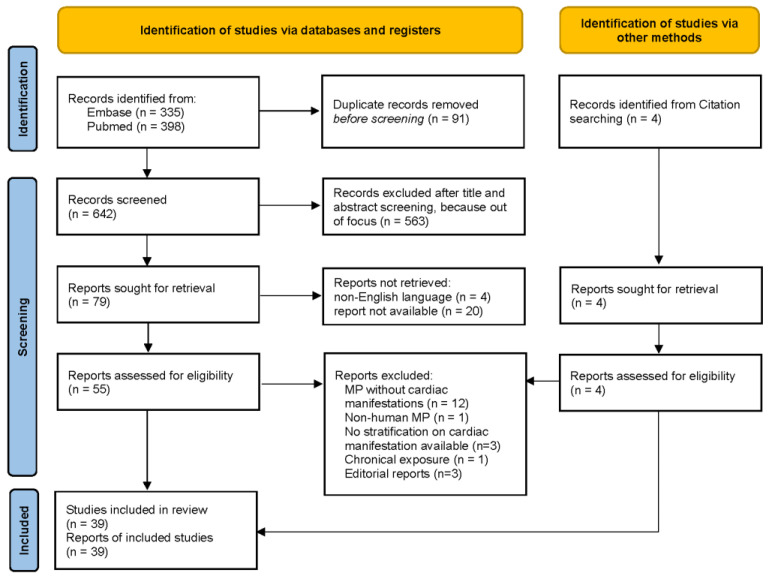
PRISMA flowchart of the present literature search. MP: mushroom poisoning.

Initially, 642 deduplicated records published from 1977 to 2023 were identified (search queries provided in the Supplementary Materials). Fifty-five reports were then retrieved, and four additional records were found by citation searching. A total of 39 studies were finally included (Figure A1 in Appendix A), of which 21 were case reports, 14 were case series, 2 were retrospective longitudinal studies, and 2 were retrospective cohort studies (Table A1 in Appendix A).

### 2.2. Patients with Cardiac Manifestations

Included studies reported data for a total of 401 patients, of which 109 (27%) as case reports or case series. We found 106 cases of cardiac manifestations related to mushroom poisoning, of which 42 were individually reported. Among these patients, sex ratio was 1.29, and median age was 52 ± 30.5 years. Cardiac manifestations occurred up to 8 days (45 ± 85 h) after mushroom ingestion.

The detailed stratification of such manifestations is presented in Table 1. The most frequently reported manifestation was an increase in cardiac enzyme concentration (57.5% of reviewed patients). Median reported CK-MB activity was 633 ± 725 UI/L (n reported = 18). Other authors reported CK-MB peak plasma concentration, which was 270.8 ± 141.8 ng/mL (n reported = 4). Some authors reported peak Troponin I concentration, which was 1.9 ± 2.21 ng/mL (n reported = 9), while others measured Troponin T, which was 1.13 ± 3.33 (n reported = 10). There were no reports in which both troponins were measured at the same time. Peak BNP plasma concentrations were reported for two patients (4000 and 5000 pg/mL), and peak nT-proBNP were reported for four patients (7414, 8230, 9961, and 32,852 pg/mL). The second most common cardiac manifestation reported was the anomaly of the ST tract at the electrocardiogram (33% of reviewed patients). It was not possible to distinguish elevations from depressions in the reported cases. Hypotension and need for amines to sustain blood pressure were almost as frequent as ST anomalies (32% of reviewed patients). Among these patients, mean arterial pressure (MAP) calculated from reported systolic and diastolic values was 70 ± 18.3 mmHg (min = 50 mmHg). Reduced cardiac output was reported for 23 patients. LVEF was 33 ± 12.5% (n reported = 18).

Detailed follow-up data were available for 58 patients. Of these, 23 completely recovered and were discharged after 7 ± 7 days, and 8 were discharged with a “partial” recovery after 20 ± 12.5 days. It was not possible to gather further information about sequelae. Eighteen patients died after 2 ± 4.25 days of hospitalization. We could gather further information on the cause of death for eleven of them: four died following acute myocarditis [10] or “severe myocardial damage” [11], three died following cardiogenic shock [12,13,14], two had multi-organ failure [15,16], one had sudden cardiac arrest [17], and one initially had hypovolemic shock but then became refractory to amine infusion [18].

### 2.3. Poisoning Severity

**Figure 1:**
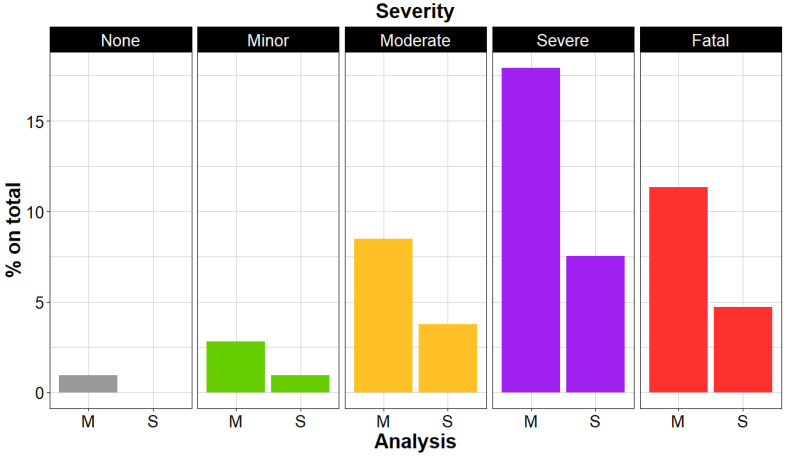
Distribution of Poisoning Severity Score. M: main analysis of patients with cardiac manifestations and individually reported data, plus two more whose death was reported (n = 44). S: sensitivity analysis on patients with cardiac manifestations and I3 or I4 causality score (n = 18).

We were able to assess the poisoning severity for the 42 patients whose individual data were reported, plus 2 more whose death was reported. Among them, one was rated with modified-PSS of “none” (modified PSS-0), three (6.8%) presented with poisoning rated as “minor” (modified PSS-1, see Section 4 for definition), nine (20.4%) with poisoning rated as “moderate” (modified PSS-2), 19 (43.2%) with poisoning rated as “severe” (modified PSS-3), and twelve (27.3%) died (“fatal poisoning”, modified PSS-4). These results are presented in Figure 1.

### 2.4. Causality Assessment

Causal relationship between cardiac manifestations and mushroom poisoning was assessed for the 42 patients whose data were individually reported. Among them, one case (2.4%) was rated with I1 (causal relationship “not excluded”), twenty-three cases (54.8%) were scored with I2 (causal relationship “possible”), eight cases (19%) were scored with I3 (causal relationship “probable”), and ten cases (23.8%) with I4 (causal relationship “very probable”).

### 2.5. Sensitivity Analysis

We included in the sensitivity analysis only the 18 patients who presented cardiac manifestations rated with I3 or I4 causality score. In this subgroup, cardiac manifestations occurred 30 ± 45 h after mushroom ingestion. Detailed manifestation stratification is provided in Table 2. Of these patients, 72.2% showed a cardiac enzyme elevation. CK-MB activity was 403 ± 588 UI/L (n reported = 5), and two CK-MB peak plasma concentrations were reported (57.5 and 291.6 ng/mL). Peak Troponin I was 1.92 ± 1.10 ng/mL (n reported = 3), and peak Troponin T was 1.22 ± 1.93 (n reported = 7). There were no cases with reported BNP, and nT-proBNP was reported for two cases (9961 and 32,852 pg/mL). Hypotension was the second most frequent condition (44.4%) as in primary analysis. On the other hand, serious conditions like cardio-respiratory arrest (44.4%) and acute heart failure (38.9%) were more frequent in the sensitivity analysis subgroup than in the primary analysis. Reported LVEF was 36 ± 12.5%. Notably, 4 cases of myocarditis out of the 7 reported overall were still present in the high causality score subgroup.

Among these patients, one (5.5%) presented with poisoning rated as “minor” (modified PSS-1), four (22.2%) with poisoning rated as “moderate” (modified PSS-2), eight (44.4%) with a poisoning rated as “severe” (modified PSS-3), and five (27.8%) died (“fatal poisoning”, modified PSS-4). As shown in Figure 1, these proportions match the ones observed in the primary analysis.

### 2.6. Involved Mushroom Species

A comprehensive list of the mushroom species identified (via direct identification and/or toxicological analysis of blood or urines) in the included cases is proposed in Table 3. Notably, *Russula*, *Trogia,* and *Amanita proxima* were the most frequently identified (in 18, 13, and 13 cases, respectively), but only *Amanita proxima* was still present in the high causality score subgroup (2 cases still present), together with 4 cases in which *Psilocybe* was identified.

Among the four cases assessed with I3 or I4 causality score following *Psilocybe* ingestion [17,19,20,21], two had symptoms after 2 h, and one after 30 h. All of them had ECG abnormalities, three had elevated cardiac enzymes, and two had cardiac arrest. Of these two, one died, and the other was discharged with sequelae. The two others were diagnosed with Tako-Tsubo syndrome.

Symptom onset was slower in I3 or I4 cases of *Amanita* poisoning [22,23,24]: 48 h for two patients and 144 h for the third one. The LVEF was substantially reduced in all three cases (32%, 25%, and 20%), despite their young age (46, 55, and 58 years, respectively). The two patients with the lowest LVEF needed an intra-aortic balloon counter-pressure (IABCP) and underwent cardiac arrest during hospitalization. All the three patients survived.

The two *Tricholoma* poisonings scored with I3 or I4 had ECG abnormalities and fatal acute heart failure [10,14]. Symptoms onset was reported only for one of them (96 h after ingestion). The only myocarditis case included in the sensitivity analysis subgroup for which the mushroom identification was available was *Tricholoma* poisoning.

## 3. Discussion

### 3.1. Clinical Presentation of Reported Poisonings

Our systematic review, originally prompted by a case of toxic myocarditis, discovered that not only other cases of myocarditis following MP have been already reported but also gave us a broader picture of all reported cases of cardiac involvement.

Unsurprisingly, the most frequently reported cardiac manifestations were the non-specific and less severe ones, such as cardiac enzyme elevation, ST segment anomalies, and hypotension. The fact that there were more patients displaying cardiac enzyme elevation than ones with ST segment anomalies could be interpreted in two ways. Either it is a “background noise” due to the non-specificity of these biomarkers in the case of coexisting rhabdomyolysis, or we could cautiously speculate that some patients experienced an ischemia-independent myocardial injury (possibly dependent on the direct myocardial effect of mushroom toxins).

Even though the median MAP of patients included in this review is above 60 mmHg, all patients which showed hypotension also received amines to sustain the arterial pressure. Moreover, some of them underwent extra-corporeal membrane oxygenation (ECMO) or IABCP. Therefore, some of the included patients definitely experienced shock during their hospital stay, even though we cannot certainly conclude on its cardiogenic etiology due to the retrospective nature of our data.

More specific conditions such as acute heart failure, myocarditis, and Tako-Tsubo syndrome have been included in our primary analysis as well as in our sensitivity analysis restricted to cases with highly probable causal relationships (I3 and I4 scores). This means that direct myocardial damage following mushroom poisoning is possible and has been reported.

While comparing the proportions of reported manifestations between the primary and the sensitivity analysis, more serious manifestations are more frequent in the second one. Furthermore, *Tricholoma* and *Psilocybe* ingestions seemed to be more lethal than *Amanita* ones, but patients with *Psilocybe* poisoning did not experience acute heart failure, differently from *Tricholoma* or *Amanita* empoisoned ones. These facts should be interpreted with extreme caution, since collected evidence is anecdotic, and it bears a non-negligible selective reporting bias.

Notably, we did not find any other reported cases of cardiac manifestations in which *Cortinarius* was identified. This may be possibly due to the long asymptomatic period of orellanian syndrome, which falsely reassures patients and prevents them from keeping mushroom samples for analysis. In addition, orellanine dosing may not be widely available, thus preventing the mushroom identification via toxin detection. The case of *Cortinarius* myocarditis that we encountered would thus be the first reported (and retrieved) so far.

### 3.2. Biochemical Bases of Reported Poisonings

Uncommon species (such as *Russula*, *Tricholoma*, or *Trogia*) were unexpectedly frequent in the reviewed literature. Nonetheless, a mechanism of cardiac toxicity for these species has been proposed elsewhere or may be supposed from their known biochemical properties.

For example, *Russula subnigricans* and *Tricholoma equestre* are known for their muscle toxicity, manifested by elevated CPK levels and even rhabdomyolysis [6,10]. The molecule involved in the toxicity of these mushrooms is the cycloprop-2-ene carboxylic acid [25]. From an anatomopathological point of view, exposure of mice to cycloprop-2-ene carboxylic acid is associated with muscle alterations like muscle fiber disorganization [10] or pericardial inflammation [26]. The precise molecular mechanism is not known, and no direct cytotoxicity was observed by cell exposure to cyclopropene carboxylic acid. Matsuura et al. hypothesized that this toxin is capable of specifically triggering the rhabdomyolysis, secondarily inducing a cascade leading to subsequent lethal poisoning. It cannot be excluded that, besides rhabdomyolysis and multi-organ failure [25], cardiomyocytic damage is another possible downstream effect.

*Trogia venenata* has been linked to sudden unexplained deaths reported in China [27]. Its specific cardiac toxicity may be caused by the non-proteinogenic amino acid 2R-amino-5-hexynoic acid and its 4S-OH derivative. This toxin was identified postmortem in intracardiac samples taken from a patient who died of sudden death after eating a meal containing *Trogia venenata* [28]. The in vivo study conducted by Zhou et al. proposed a toxic mechanism depending on hypoglycemia, possibly caused by an inhibition of lipid beta-oxidation [28].

Interestingly, the 2-amino-5-hexynoic acid, as an amino acid with C5 side chain and SP-terminal poly-unsaturation, presents a very high chemical similarity with 2-aminohexa-4,5-dienoic acid (also called allenoic norleucine), which could be involved in the toxicity of amatoxin-free *Amanita*-like *A. proxima*. The presence of this toxin has been demonstrated in *A. smithiana* [29] or *A. boudieri* [29,30] and is discussed for *A. proxima* [16,29,30,31].

The cardiotoxicity of *Psilocybe* mushrooms could be explained by the pharmacological properties of some of their psychoactive compounds, psilocybin and psilocin: like many indole-derivatives, they possess well-known serotoninergic properties that can induce tachycardia, vasoconstriction, particularly coronary vasoconstriction, and platelet aggregation [19]. Consequently, the pro-ischemic effect has been proposed as a possible toxicity mechanism for psylocibin derivatives [32].

### 3.3. Strengths and Limits of Our Review

The strength of our work is based on its solid methodological bases. Thanks to our systematic approach to the literature review, as well as the preregistration of protocol and the sharing of data and code, we ensured the reproducibility of our results. All critical steps of the review, as well as the data extraction and appraisal, were performed by two researchers, ensuring the robustness of the processing phase. To assess the severity and the causal relationship, we adapted two tools well validated and broadly used in clinical toxicology practice.

Nonetheless, in spite of our efforts to broadly and systematically screen the available literature, we acknowledge that this review may not be exhaustive. In addition, as often retrospective research does, our study could bear some biases. First, since the main source of the reviewed evidence is made of case reports and case series, rather than cohort studies, our synthesis is almost surely affected by a selective reporting bias, skewing the proportion of reported cases towards higher clinical severity or rarer mushroom species involved. The total sample of included patients is too narrow to allow a good inference power to the general population, as the reader can see by the broad confidence intervals of proportions proposed herein. Furthermore, there is substantial clinical heterogeneity in the reviewed cases that we could not mitigate in our analysis due to the limitation of retrospective data collection. Consequently, the proposed quantitative synthetic measures exhibit high variance and cannot be promptly interpreted.

### 3.4. Conclusions

In the present study, we applied the state-of-the-art methods to systematically synthetize the available knowledge on clinical manifestations and possible biochemical explanations of cardiac toxicity resulting from mushroom poisoning. Despite the unmitigable selection bias, we showed that this type of toxicity, albeit rare, has been documented, and for some of the reported cases, we retrospectively assessed a strong causal relationship between the mushroom intake and the cardiac involvement.

This study can be already of hand to all clinical toxicologists who look for previous evidence to appraise the clinical condition of their patients, like we were looking for when we encountered a case of myocarditis after mushroom ingestion.

### 3.5. Future Directions

In future, this evidence needs to be strengthened with better research methodologies. Exhaustive retrospective queries of Poison Control Center databases could mitigate the selection bias, thus providing more reliable proportions of involved mushrooms and clinical presentations. Prospective cohort studies could help us to characterize the clinical manifestations with lesser confounding variables and misclassifications. Finally, as translational researchers, we hope for future bedside-to-bench approaches, leading to new studies on the cardiotoxic effects of mushroom toxins.

## 4. Methods

### 4.1. Literature Search

We searched Embase and MEDLINE databases for reports published in English or French language before 16 February 2023. Research queries are provided in Supplementary Materials. Furthermore, we examined the references cited in the retrieved articles, adding relevant citations to the overall search results.

The check for duplicates was partially automatized through the built-in functions of EndNote X9 (Clarivate Analytics, London, UK) and Mendeley 2.75 (Mendeley, London, UK). After the removal of duplicates, we screened records by abstract, and then, full texts were reading. The article selection process was independently conducted by M.B. and G.B., and discordances were solved by consensus at the end of process. The PRISMA flowchart of the literature review is showed in Figure A1. The list of reports sought for retrieval and reports assessed for eligibility is provided in the online repository of this project (Open Science Framework number: A8QFB).

### 4.2. Inclusion Criteria

Studies finally included reported data on humans who had at least one of the following cardiac manifestations:

- New-onset arrhythmia or other electrocardiogram abnormalities;
- Alteration in left-ventricle ejection fraction (LVEF) at cardiac ultrasound imaging;
- Elevated troponin or creatine phosphokinase MB (CK-MB) plasma concentrations;
- Hypotension (<90/60 mmHg) or need for amine administration to sustain blood pressure;
- Cardio-pulmonary arrest.

### 4.3. Data Extraction and Processing

For each patient (or group of patients), the following data were collected by M.B. throughout full-text reading: ingested mushroom species, type and onset delay of cardiac symptoms, type of critical care needed (amine administration, intra-aortic balloon counter-pulsation (IABCP), or extra-corporeal membrane oxygenation (ECMO)), and follow-up data.

To describe the poisoning severity, we assessed a “modified” Poisoning Severity Score (PSS) for each individually reported patient, adapting the score originally developed by the European Association of Poisons Centres and clinical toxicologists. Such scoring method was evaluated by Persson et al. [33] and showed an 80% overall concordance between evaluators (73% in *Amanita* poisoning cases). The PSS has five levels, named as follows: “fatal” (PSS-4), “severe” (PSS-3), “moderate” (PSS-2), “minor” (PSS-1), and “none” (PSS-0).

In addition, G.B. and M.B. independently assessed the strength of the causal relationship of the individually reported cases using the algorithm validated by the French Toxicovigilance Coordination Committee. The score has six levels, named as follows: “very probable” (I4), “probable” (I3), “possible” (I2), “not excluded” (I1), “excluded” (I0), and “not evaluable” (Ii). Further details on the score calculation method are provided in the Supplementary Materials (Tables from S1 to S6 contain the detail for the calculation of each axe’s score, while Table S7 contains the overall score calculation matrix). Discordances were solved by consensus at the end of process.

Summary statistics for age and cardiac enzyme plasma concentration were retrieved and pooled to individually reported measures, according to the method described in the Cochrane Handbook for Systematic Reviews [34], to obtain the overall estimation. If not differently stated, data are herein always reported as median ± interquartile range (normality of distributions could not be tested). Proportions are provided with a 95% confidence interval calculated with the Clopper–Pearson exact method. Data were processed with Microsoft Excel 365 (Redmond, WA, USA) and R 4.3.1.

### 4.4. Outcomes

The primary outcome was the overall number of patients showing cardiac manifestations, stratified on each cardiac manifestation. Secondary outcomes were the severity and the strength of the causal relationship of each poisoning case. Sensitivity analyses included only patients with I3 (“probable”) or I4 (“very probable”) causal relationship score.

### 4.5. Quality Assessment

The research protocol was internally approved on 3 December 2021 and registered on the Open Science Framework on 14 April 2022 (registration number: A8QFB). A second version of the research protocol, amended to extend the timespan of the literature review and to optimize the statistical methods, was internally approved and registered on 1 February 2023. The quality of review has been assessed according to PRISMA guidelines [35] (Supplementary Materials Table S8).

## Figures and Tables

**Table 1:** Stratification of cardiac manifestations in all included patients. Conditions have been sorted on their clinical severity, rather than alphabetically. One patient may have displayed more than one condition. 95% confidence intervals of proportions are provided.

	n	%	95% CI
Patients with cardiac manifestations	106	100.00	
Cardiac enzyme increase	61	57.55	47.57–67.09
ST modifications	35	33.02	24.19–42.82
Hypotension	34	32.08	23.34–41.84
Decreased LVEF	23	21.70	14.28–30.76
Acute heart failure	18	16.98	10.39–25.5
Myocarditis	7	6.60	2.7–13.13
Tako-Tsubo syndrome	3	2.83	0.59–8.05
Ventricular tachycardia	14	13.21	7.41–21.17
Use of ECMO or IABCP	5	4.72	1.55–10.67
Need of amines to sustain BP	34	32.08	23.34–41.84
Cardio-respiratory arrest	16	15.09	8.88–23.35
Death	18	16.98	10.39–25.5

BP: blood pressure; ECMO: extra-corporeal membrane oxygenation; IABCP: intra-aortic balloon counter-pressure; and LVEF: left-ventricle ejection fraction.

**Table 2:** Stratification of cardiac manifestations in patients with I3 or I4 causality score (sensitivity analysis). Conditions were sorted based on their clinical severity, rather than alphabetically. One patient may have displayed more than one condition. 95% confidence intervals of proportions are provided.

	n	%	95% CI
Patients with cardiac manifestations and I3 or I4 causality score	18	100.00	
Cardiac enzyme increase	13	72.22	46.52–90.31
ST modifications	7	38.89	17.3–64.25
Hypotension	8	44.44	21.53–69.24
Decreased LVEF	6	33.33	13.34–59.01
Acute heart failure	7	38.89	17.3–64.25
Myocarditis	4	22.22	6.41–47.64
Tako-Tsubo syndrome	2	11.11	1.38–34.71
Ventricular tachycardia	5	27.78	9.69–53.48
Use of ECMO or IABCP	2	11.11	1.38–34.71
Need of amines to sustain BP	5	27.78	9.69–53.48
Cardio-respiratory arrest	8	44.44	21.53–69.24
Death	5	27.78	9.69–53.48

BP: blood pressure; ECMO: extra-corporeal membrane oxygenation; IABCP: intra-aortic balloon counter-pressure; and LVEF: left-ventricle ejection fraction.

**Table 3:** Mushroom species involved in the cases with cardiac manifestations.

Mushroom Genus	Total Cases (n = 106)	I3 and I4 Cases (n = 18)
*Amanita*	23 ^a^	3 ^b^
*Conocybe*	1	1
*Coprinus*	1	0
*Lepiota*	1 ^c^	1 ^c^
*Pleurotes*	1	1
*Psilocybe*	5	4
*Rubinoboletus*	1	1
*Russula*	18	0
*Tricholoma*	8	2
*Trogia*	13	0
Not identified	34	5

a: of which, 9 *phalloides*, 13 *proxima,* and 1 not reported. b: of which, 2 *proxima* and 1 not reported. c: *Lepiota chlorophyllum*.

## Data Availability

Publicly available datasets were analyzed in this study. These data, as well as the R code used, can be found on the OSF repository for this project: https://osf.io/a8qfb/ (accessed on 7 June 2024).

## Supplementary Materials

The following supporting information can be downloaded at: https://www.mdpi.com/article/10.3390/toxins16060265/s1, French Toxicovigilance Coordination Committee causality assessment algorithm. Causality assessment in the present study. Table S1: Exposure score (E) levels; Table S2: Symptoms score (S) levels; Table S3: Chronology score (C) levels; Table S4: Objective diagnostic elements score (L) levels; Table S5: Differential diagnosis score (D) levels; Table S6: Literature agreement score (B) levels; Table S7: Overall causality matrix; Table S8: PRISMA checklist for systematic reviews.

## Appendix A

Further details on the literature search steps followed (Figure A1) and on the included articles (Table A1) are presented in this section.

**Table A1:** List of included articles alphabetically sorted.

Reference	Report Type	Country	Patients	Patients with Cardiac Manifestations
Aleman [36]	CR	The Netherlands	1	1
Altintepe [22]	CS	Turkey	3	3
Anand [12]	CS	Poland	4	1
Aygul [37]	CR	Turkey	1	1
Bedry [10]	CS	France	12	3
Besancon [38]	CS	France	2	2
Borowiak [19]	CR	Poland	1	1
Avci [39]	CR	Turkey	1	1
Caley [40]	CR	The United Kingdom	1	1
Cho [13]	CR	South Korea	1	1
Deng [11]	L, R	China	58	1
Erenler [41]	L, R	Turkey	175	22
Feinfeld [15]	CS	The USA	4	1
Frass [42]	CS	Bulgaria	2	2
Kalcik [43]	CS	Turkey	4	1
Karakoc [44]	CR	Turkey	1	1
Karvellas [45]	CO, R	The USA	18	5
Kessler [23]	CR	Israel	1	1
Kotts [20]	CR	The United Kingdom	1	1
Koyuncu [46]	CS	Turkey	2	1
Laubner [14]	CS	Lithuania	4	4
Lee [47]	CS	Taiwan	9	1
Leonardi [16]	CS	Italy	8	8
Li S. [48]	CR	China	1	1
Li Y. [49]	CR	China	1	1
Lim [17]	CR	New Zealand	1	1
Lin [50]	CS	China	7	1
Machado [24]	CR	France	1	1
Min [51]	CS	China	4	1
Nef [21]	CR	Germany	1	1
Palma [52]	CR	Italy	1	1
Pauli [18]	CR	Australia	1	1
Ponsiglione [8]	CR	Italy	1	1
Qin [53]	CR	China	1	1
Tepetam [54]	CR	Turkey	1	1
Trakulsrichai [55]	CO, R	Thailand	41	15
Unverir [56]	CR	Turkey	1	1
Vallianou [9]	CR	Greece	1	1
Zhao [57]	CS	China	23	13

CO: cohort; CR: case report; CS: case series; L: longitudinal; and R: retrospective.
