# Supplementary material for: Mushroom Poisoning-Related Cardiac Toxicity: A Case Report and Systematic Review
Source: Toxins (Basel). 2024 Jun 10;16(6):265. doi: 10.3390/toxins16060265 (PMC11209194; doi:10.3390/toxins16060265)
Supplement: Supplementary file 1 [file toxins-16-00265-s001.zip › toxins-2980820-supplementary.pdf]

toxins

MDPI

Supplementary Materials: Mushroom Poisoning-Related Cardiac Toxicity: A Case Report and Systematic Review

Giuseppe Balice, Maxime Boksebeld, Quentin Barrier, Sara Boccalini, Behrouz Kassai-Koupai, Nathalie Paret and Guillaume Grenet

1. French Toxicovigilance Coordination Committee causality assessment algorithm

Causality relationship between a clinical manifestation and a poisoning event is described by the six following axes: exposition (E, Table S1), symptoms (S, Table S2), chronology (C, Table S3), presence of objective diagnostic elements (L, Table S4), ruling out of other differential diagnoses (D, Table S5), and agreement with literature (B, Table S6).

Table S1. Exposure score (E) levels.

Score	Level name	Criteria
E2	Very probable	Exposure confirmed by objective elements or witnesses
E1	Possible	No objective element available to confirm or exclude exposure
E0	Excluded	Objective elements allow to exclude an exposure

Table S2. Symptoms score (S) levels.

Score	Level name	Criteria
S1	Present	Clinical signs observed or symptoms reported
S0	Absent	No clinical signs nor symptoms

Table S3. Chronology score (C) levels.

Score	Level name	Criteria
C2	Suggesting	Signs or symptoms appeared within the toxicokinetic peak concentration time OR after each rechallenge
C1	Compatible	Signs or symptoms appeared after toxicokinetic peak concentration time OR persisted after exposition end OR no chronology data available
C0	Incompatible	Signs or symptoms appeared before the exposition OR at a time judged too early with regard to toxicodynamics

Table S4. Objective diagnostic element score (L) levels.

Score	Level name	Criteria
L2	Probative elements	Specific test resulted in positive OR agreement between observed and predicted TK/TD profiles OR agreement between topology of local effect and topology of exposition
L1	No probative elements	No specific test performed
L0	Refuting elements	Specific test resulted in negative OR disagreement between observed and predicted TK/TD profiles

Table S5. Differential diagnosis score (D) levels.

Score	Level name	Criteria
D2	Excluded	All differential diagnoses ruled out after the workup
D1	Absent	No differential diagnoses proposed OR no other differential diagnosis explored during workup
D0	Retained	A differential diagnosis retained at workup

Table S6. Literature agreement score (B) levels.

Score	Level name	Criteria
B2	Relationship probable	Strong evidence in humans available OR weak evidence in human and strong evidence in animal models available
B1	Relationship possible	Strong evidence in animal models available
B0	Never described	No evidence available yet

The overall imputability score has six levels, named as follows: very probable (I4), probable (I3), possible (I2), not excluded (I1), excluded (I0), and not evaluable (Ii). The score is calculated using the matrix in Table S7.

Table S7. Overall imputability matrix.

Exposure	Symptoms	Chronology	Objective diagnostic elements	Differential diagnosis	Bibliography	Overall imputability
E2	S1	C2	L2	D2	B2	I4
E2	S1	C2	L2	D2	B1	I4
E2	S1	C2	L2	D1	B2	I4
E2	S1	C2	L2	D1	B1	I4
E2	S1	C1	L2	D2	B2	I4
E2	S1	C1	L2	D2	B1	I4
E2	S1	C1	L2	D1	B2	I4
E2	S1	C1	L2	D1	B1	I4
E1	S1	C2	L2	D2	B2	I4
E1	S1	C2	L2	D2	B1	I4
E1	S1	C2	L2	D1	B2	I4
E1	S1	C1	L2	D2	B2	I3
E2	S1	C2	L2	D2	B0	I3
E2	S1	C2	L2	D1	B0	I3
E2	S1	C2	L1	D2	B2	I3
E2	S1	C2	L1	D2	B1	I3
E2	S1	C2	L1	D1	B2	I3
E2	S1	C1	L2	D2	B0	I3
E2	S1	C1	L2	D1	B0	I3
E2	S1	C1	L1	D2	B2	I3
E2	S1	C1	L1	D2	B1	I3
E2	S1	C1	L1	D1	B2	I3
E1	S1	C2	L2	D2	B0	I3
E1	S1	C2	L2	D1	B1	I3
E1	S1	C2	L1	D2	B2	I3
E1	S1	C2	L1	D2	B1	I3
E1	S1	C2	L1	D2	B0	I3
E1	S1	C2	L1	D1	B2	I3
E1	S1	C1	L2	D2	B1	I3
E1	S1	C1	L2	D2	B0	I3
E1	S1	C1	L2	D1	B2	I3
E1	S1	C1	L1	D2	B2	I3
E1	S1	C1	L1	D2	B1	I3
E1	S1	C1	L1	D1	B2	I2
E1	S1	C1	L1	D2	B0	I2
E1	S1	C1	L2	D1	B0	I2
E1	S1	C1	L2	D1	B1	I2

Table S7. Overall imputability matrix (continues)

Exposure	Symptoms	Chronology	Objective diagnostic elements	Differential diagnosis	Bibliography	Overall imputability
E1	S1	C2	L1	D1	B0	I2
E1	S1	C2	L2	D1	B0	I2
E2	S1	C2	L1	D1	B1	I2
E2	S1	C2	L1	D2	B0	I2
E2	S1	C1	L1	D1	B1	I1
E1	S1	C1	L1	D1	B1	I1
E1	S1	C1	L1	D0	-	I1
E1	S1	C1	L1	D1	B0	I1
E1	S1	C1	L2	D0	-	I1
E1	S1	C2	L1	D0	-	I1
E1	S1	C2	L1	D1	B0	I1
E1	S1	C2	L2	D0	-	I1
E2	S1	C1	L1	D0	-	I1
E2	S1	C1	L1	D1	B0	I1
E2	S1	C1	L2	D0	-	I1
E2	S1	C2	L1	D0	-	I1
E2	S1	C2	L1	D1	B0	I1
E2	S1	C2	L2	D0	-	I1
E0	-	-	-	-	-	I0
-	-	C0	-	-	-	I0
-	-	-	L0	-	-	I0
-	S0	-	-	-	-	Ii

2. Causality assessment in the present study

According to the original French Toxicovigilance Coordination Committee criteria, we systematically assigned the following scores to all cases included in the present study: presence of symptoms (S1), very probable exposure (E2), and possible relationship according to literature (B1).

Objective diagnostic element (L) axis was evaluated based on the presence of toxins. Differential diagnosis (D) axis was scored according to the original criteria.

We modified the chronology (C) criteria so that signs or symptoms appeared within 7 days from exposure were rated as suggesting chronology (C2), and signs and symptoms appeared from the 8th day after exposure were rated as compatible chronology (C1).

3. Quality assessment of the present systematic review

Table S8. PRISMA checklist for systematic reviews.

Section and Topic	Item #	Checklist item	Lines
**INTRODUCTION**			
Rationale	3	Describe the rationale for the review in the context of existing knowledge.	56-61
Objectives	4	Provide an explicit statement of the objective(s) or question(s) the review addresses.	62-65
**METHODS**			
Eligibility criteria	5	Specify the inclusion and exclusion criteria for the review and how studies were grouped for the syntheses.	230-238
Information sources	6	Specify all databases, registers, websites, organizations, reference lists and other sources searched or consulted to identify studies. Specify the date when each source was last searched or consulted.	219-223
Search strategy	7	Present the full search strategies for all databases, registers and websites, including any filters and limits used.	S47-S101
Selection process	8	Specify the methods used to decide whether a study met the inclusion criteria of the review, including how many reviewers screened each record and each report retrieved, whether they worked independently, and if applicable, details of automation tools used in the process.	224-228
Data collection process	9	Specify the methods used to collect data from reports, including how many reviewers collected data from each report, whether they worked independently, any processes for obtaining or confirming data from study investigators, and if applicable, details of automation tools used in the process.	241-244
Data items	10a	List and define all outcomes for which data were sought. Specify whether all results that were compatible with each outcome domain in each study were sought (e.g., for all measures, time points, analyses), and if not, the methods used to decide which results to collect.	241-251
	10b	List and define all other variables for which data were sought (e.g., participant and intervention characteristics, funding sources). Describe any assumptions made about any missing or unclear information.	Not performed
Study risk of bias assessment	11	Specify the methods used to assess risk of bias in the included studies, including details of the tool(s) used, how many reviewers assessed each study and whether they worked independently, and if applicable, details of automation tools used in the process.	Deemed not necessary
Effect measures	12	Specify for each outcome the effect measure(s) (e.g., risk ratio, mean difference) used in the synthesis or presentation of results.	254-258
Synthesis methods	13a	Describe the processes used to decide which studies were eligible for each synthesis (e.g., tabulating the study intervention characteristics and comparing against the planned groups for each synthesis (item #5)).	Deemed not necessary
	13b	Describe any methods required to prepare the data for presentation or synthesis, such as handling of missing summary statistics, or data conversions.	252-254

Section and Topic	Item #	Checklist item	Lines
	13c	Describe any methods used to tabulate or visually display results of individual studies and syntheses.	Deemed not necessary
	13d	Describe any methods used to synthesize results and provide a rationale for the choice(s). If meta-analysis was performed, describe the model(s), method(s) to identify the presence and extent of statistical heterogeneity, and software package(s) used.	Deemed not necessary
	13e	Describe any methods used to explore possible causes of heterogeneity among study results (e.g., subgroup analysis, meta-regression).	Deemed not necessary
	13f	Describe any sensitivity analyses conducted to assess robustness of the synthesized results.	263-264
Reporting bias assessment	14	Describe any methods used to assess risk of bias due to missing results in a synthesis (arising from reporting biases).	Deemed not necessary
Certainty assessment	15	Describe any methods used to assess certainty (or confidence) in the body of evidence for an outcome.	Deemed not necessary
**RESULTS**			
Study selection	16a	Describe the results of the search and selection process, from the number of records identified in the search to the number of studies included in the review, ideally using a flow diagram.	95-99, Figure A1
	16b	Cite studies that might appear to meet the inclusion criteria, but which were excluded, and explain why they were excluded.	Figure A1
Study characteristics	17	Cite each included study and present its characteristics.	Table A1
Risk of bias in studies	18	Present assessments of risk of bias for each included study.	Deemed not necessary
Results of individual studies	19	For all outcomes, present, for each study: (a) summary statistics for each group (where appropriate) and (b) an effect estimate and its precision (e.g., confidence/credible interval), ideally using structured tables or plots.	Table 1
Results of syntheses	20a	For each synthesis, briefly summarize the characteristics and risk of bias among contributing studies.	Deemed not necessary
	20b	Present results of all statistical syntheses conducted. If meta-analysis was done, present for each the summary estimate and its precision (e.g., confidence/credible interval) and measures of statistical heterogeneity. If comparing groups, describe the direction of the effect.	102-147
	20c	Present results of all investigations of possible causes of heterogeneity among study results.	Deemed not necessary

Section and Topic	Item #	Checklist item	Lines
	20d	Present results of all sensitivity analyses conducted to assess the robustness of the synthesized results.	140-146, Tables 2 and 3
Reporting biases	21	Present assessments of risk of bias due to missing results (arising from reporting biases) for each synthesis assessed.	Not performed
Certainty of evidence	22	Present assessments of certainty (or confidence) in the body of evidence for each outcome assessed.	Tables 1 and 2
**DISCUSSION**			
Discussion	23a	Provide a general interpretation of the results in the context of other evidence.	167-172
	23b	Discuss any limitations of the evidence included in the review.	208-211
	23c	Discuss any limitations of the review processes used.	208-211
	23d	Discuss implications of the results for practice, policy, and future research.	212-216
**OTHER INFORMATION**			
Registration and protocol	24a	Provide registration information for the review, including register name and registration number, or state that the review was not registered.	267-270
	24b	Indicate where the review protocol can be accessed, or state that a protocol was not prepared.	267-270
	24c	Describe and explain any amendments to information provided at registration or in the protocol.	267-270
Support	25	Describe sources of financial or non-financial support for the review, and the role of the funders or sponsors in the review.	284
Competing interests	26	Declare any competing interests of review authors.	289
Availability of data, code and other materials	27	Report which of the following are publicly available and where they can be found: template data collection forms; data extracted from included studies; data used for all analyses; analytic code; any other materials used in the review.	287-288

4. Query for EMBASE search

('mycetism'/exp OR mycetism OR (('mushroom'/exp OR mushroom) AND poisoned) OR (('mushrooms'/exp OR mushrooms) AND poisoned) OR (('mushroom'/exp OR mushroom) AND ('poisoning'/exp OR poisoning)) OR (('mushrooms'/exp OR mushrooms) AND ('poisoning'/exp OR poisoning)) OR (('mushroom'/exp OR mushroom) AND ('ingestion'/exp OR ingestion)) OR (('mushrooms'/exp OR mushrooms) AND ('ingestion'/exp OR ingestion)) OR mushroom* OR 'amanita'/exp OR amanita OR 'cortinarius'/exp OR cortinarius OR galerina OR lepiota OR gyromitra OR conocybe OR clitocybe OR 'inocybe'/exp OR inocybe OR 'russula'/exp OR russula OR trogia OR 'psilocybe'/exp OR psilocybe OR entoloma OR amanitin* OR 'orellanine'/exp OR orellanine) AND ('myocarditis'/exp OR myocarditis OR (('cardiac'/exp OR cardiac) AND event) OR (cardiogenic AND ('shock'/exp OR shock)) OR (myocardial AND ('failure'/exp OR failure)) OR (('heart'/exp OR heart) AND disease*) OR 'arrythmia'/exp OR arrythmia OR 'cardiomyopathy'/exp OR cardiomyopathy OR (('cardiac'/exp OR cardiac) AND insufficiency) OR (('heart'/exp OR heart) AND failure*) OR (('cardiac'/exp OR cardiac) AND ('arrest'/exp OR arrest)))

5. Query for MEDLINE search

("mushroom poisoning"[MeSH Terms] OR ("mushroom"[All Fields] AND "poisoning"[All Fields]) OR "mushroom poisoning"[All Fields] OR "mycetism"[All Fields] OR (("agaricales"[MeSH Terms] OR "agaricales"[All Fields] OR

"mushroom"[All Fields] OR "mushrooms"[All Fields]) AND ("poisoned"[All Fields] OR "poisoning"[MeSH Terms] OR "poisoning"[All Fields] OR "poisonings"[All Fields] OR "poisoning"[MeSH Subheading] OR "poisonous"[All Fields] OR "poisons"[Pharmacological Action] OR "poisons"[MeSH Terms] OR "poisons"[All Fields] OR "poison"[All Fields])) OR (("agaricales"[MeSH Terms] OR "agaricales"[All Fields] OR "mushroom"[All Fields] OR "mushrooms"[All Fields]) AND ("poisoned"[All Fields] OR "poisoning"[MeSH Terms] OR "poisoning"[All Fields] OR "poisonings"[All Fields] OR "poisoning"[MeSH Subheading] OR "poisonous"[All Fields] OR "poisons"[Pharmacological Action] OR "poisons"[MeSH Terms] OR "poisons"[All Fields] OR "poison"[All Fields])) OR ("mushroom poisoning"[MeSH Terms] OR ("mushroom"[All Fields] AND "poisoning"[All Fields]) OR "mushroom poisoning"[All Fields]) OR (("agaricales"[MeSH Terms] OR "agaricales"[All Fields] OR "mushroom"[All Fields] OR "mushrooms"[All Fields]) AND ("poisoned"[All Fields] OR "poisoning"[MeSH Terms] OR "poisoning"[All Fields] OR "poisonings"[All Fields] OR "poisoning"[MeSH Subheading] OR "poisonous"[All Fields] OR "poisons"[Pharmacological Action] OR "poisons"[MeSH Terms] OR "poisons"[All Fields] OR "poison"[All Fields])) OR (("agaricales"[MeSH Terms] OR "agaricales"[All Fields] OR "mushroom"[All Fields] OR "mushrooms"[All Fields]) AND ("eating"[MeSH Terms] OR "eating"[All Fields] OR "ingest"[All Fields] OR "ingested"[All Fields] OR "ingesting"[All Fields] OR "ingestion"[All Fields] OR "ingestions"[All Fields] OR "ingesters"[All Fields] OR "ingests"[All Fields])) OR (("agaricales"[MeSH Terms] OR "agaricales"[All Fields] OR "mushroom"[All Fields] OR "mushrooms"[All Fields]) AND ("eating"[MeSH Terms] OR "eating"[All Fields] OR "ingest"[All Fields] OR "ingested"[All Fields] OR "ingesting"[All Fields] OR "ingestion"[All Fields] OR "ingestions"[All Fields] OR "ingesters"[All Fields] OR "ingests"[All Fields])) OR "mushroom*"[All Fields] OR ("amanita"[MeSH Terms] OR "amanita"[All Fields] OR "amanitas"[All Fields]) OR ("cortinarius"[MeSH Terms] OR "cortinarius"[All Fields]) OR "Galerina"[All Fields] OR "Lepiota"[All Fields] OR "Gyromitra"[All Fields] OR "Conocybe"[All Fields] OR "Clitocybe"[All Fields] OR "Inocybe"[All Fields] OR "Russula"[All Fields] OR "Trogia"[All Fields] OR ("psilocybe"[MeSH Terms] OR "psilocybe"[All Fields] OR "psilocybes"[All Fields]) OR "Entoloma"[All Fields] OR "amanitin*"[All Fields] OR ("orellanine"[Supplementary Concept] OR "orellanine"[All Fields])) AND ("myocardic"[All Fields] OR "myocarditis"[MeSH Terms] OR "myocarditis"[All Fields] OR "myocarditides"[All Fields] OR ("cardiovascular diseases"[MeSH Terms] OR ("cardiovascular"[All Fields] AND "diseases"[All Fields]) OR "cardiovascular diseases"[All Fields] OR ("cardiac"[All Fields] AND "event"[All Fields]) OR "cardiac event"[All Fields]) OR ("shock, cardiogenic"[MeSH Terms] OR ("shock"[All Fields] AND "cardiogenic"[All Fields]) OR "cardiogenic shock"[All Fields] OR ("cardiogenic"[All Fields] AND "shock"[All Fields])) OR ("heart failure"[MeSH Terms] OR ("heart"[All Fields] AND "failure"[All Fields]) OR "heart failure"[All Fields] OR ("myocardial"[All Fields] AND "failure"[All Fields]) OR "myocardial failure"[All Fields]) OR (("heart"[MeSH Terms] OR "heart"[All Fields] OR "hearts"[All Fields] OR "heart s"[All Fields]) AND "disease*"[All Fields]) OR ("arrhythmias, cardiac"[MeSH Terms] OR ("arrhythmias"[All Fields] AND "cardiac"[All Fields]) OR "cardiac arrhythmias"[All Fields] OR "arrythmia"[All Fields] OR "arrythmias"[All Fields]) OR ("cardiomyopathie"[All Fields] OR "cardiomyopathies"[MeSH Terms] OR "cardiomyopathies"[All Fields] OR "cardiomyopathy"[All Fields]) OR ("heart failure"[MeSH Terms] OR ("heart"[All Fields] AND "failure"[All Fields]) OR "heart failure"[All Fields] OR ("cardiac"[All Fields] AND "insufficiency"[All Fields]) OR "cardiac insufficiency"[All Fields]) OR (("heart"[MeSH Terms] OR "heart"[All Fields] OR "hearts"[All Fields] OR "heart s"[All Fields]) AND "failure*"[All Fields]) OR ("heart arrest"[MeSH Terms] OR ("heart"[All Fields] AND "arrest"[All Fields]) OR "heart arrest"[All Fields] OR ("cardiac"[All Fields] AND "arrest"[All Fields]) OR "cardiac arrest"[All Fields]))
